# New York State Society Report

**Published:** 1884-06

**Authors:** 


### NEW YORK STATE SOCIETY REPORT.

The sixteenth annual meeting of the Dental Society of the State of New York was of more than usual interest. The attendance was good, and some of the papers read were of permanent value. We are unable to commence the publication of a stenographic report this month, but next month will present the prize-essay of Dr. Frank Abbott, together with a portion of the report, and this will be followed by the remainder as fast as space will permit.

A full stenographic report of the meeting of the Illinois State Dental Society is necessarily deferred also, because of the illness of the reporter, who is not able to write out his notes.

